# Gut Microbiota, Bacterial Translocation, and Stroke: Current Knowledge and Future Directions

**DOI:** 10.3390/biomedicines12122781

**Published:** 2024-12-06

**Authors:** Cristina Granados-Martinez, Nuria Alfageme-Lopez, Manuel Navarro-Oviedo, Carmen Nieto-Vaquero, Maria Isabel Cuartero, Blanca Diaz-Benito, Maria Angeles Moro, Ignacio Lizasoain, Macarena Hernandez-Jimenez, Jesus Miguel Pradillo

**Affiliations:** 1 Department of Pharmacology and Toxicology, School of Medicine, University Complutense of Madrid, 28040 Madrid, Spain; crisgran@ucm.es (C.G.-M.); nualfa01@ucm.es (N.A.-L.); manuna02@ucm.es (M.N.-O.); carmen.nieto@cnic.es (C.N.-V.); maricuar@ucm.es (M.I.C.); bldiaz03@ucm.es (B.D.-B.); 2 Research Institute Hospital 12 de Octubre, 28041 Madrid, Spain; ignacio.lizasoain@med.ucm.es; 3 Neurovascular Pathophysiology, Cardiovascular Risk Factor and Brain Health Program, Centro Nacional de Investigaciones Cardiovasculares (CNIC), 28029 Madrid, Spain; 4 AptaTargets S.L. Avda. Cardenal Herrera Oria 298, 28035 Madrid, Spain

**Keywords:** stroke, ischemic stroke, hemorrhagic stroke, dysbiosis, leaky gut, bacterial translocation, stroke associated infections, treatment

## Abstract

Stroke is one of the most devastating pathologies in terms of mortality, cause of dementia, major adult disability, and socioeconomic burden worldwide. Despite its severity, treatment options remain limited, with no pharmacological therapies available for hemorrhagic stroke (HS) and only fibrinolytic therapy or mechanical thrombectomy for ischemic stroke (IS). In the pathophysiology of stroke, after the acute phase, many patients develop systemic immunosuppression, which, combined with neurological dysfunction and hospital management, leads to the onset of stroke-associated infections (SAIs). These infections worsen prognosis and increase mortality. Recent evidence, particularly from experimental studies, has highlighted alterations in the microbiota–gut–brain axis (MGBA) following stroke, which ultimately disrupts the gut flora and increases intestinal permeability. These changes can result in bacterial translocation (BT) from the gut to sterile organs, further contributing to the development of SAIs. Given the novelty and significance of these processes, especially the role of BT in the development of SAIs, this review summarizes the latest advances in understanding these phenomena and discusses potential therapeutic strategies to mitigate them, ultimately reducing post-stroke complications and improving treatment outcomes.

## 1. Introduction

Stroke is one of the most devastating pathologies in terms of mortality, cause of dementia, major adult disability, and socioeconomic burden worldwide. In the European Union, stroke affects ≈ 1.1 million inhabitants per year being the second cause of death and a leading cause of disability in adults [1]. Two main types of stroke can be distinguished: ischemic stroke (IS) and hemorrhagic stroke (HS). IS, which accounts for about 85% of cases, is caused by the blockage of a cerebral blood vessel, while HS, responsible for the remaining 15%, is caused by bleeding in or around the brain and tends to be more severe [2]. Despite the significant impact of this pathology, its treatment remains highly insufficient. The medical management of stroke begins with a rapid diagnosis, which involves imaging techniques such as computed tomography (CT) or magnetic resonance imaging (MRI) to differentiate between IS and HS, locate the lesion, and assess its extent [3]. After diagnosis, the next step is the administration of appropriate treatment, which can be mechanical (e.g., craniectomy or endovascular therapy, depending on the type of stroke) or pharmacological. For IS, pharmacological treatment typically involves clot fibrinolysis using Alteplase or Tenecteplase, which is effective within the first 4.5 to 9 h after symptom onset due to their potential side effects, and only in patients who are well diagnosed with advanced neuroimaging. In some specific cases, mechanical clot removal via endovascular thrombectomy can be performed up to 24 h after symptom onset [4]. However, these therapies are only available to a small percentage of patients [5]. For HS, despite numerous preclinical and clinical trials over the past decades, no pharmacological treatments have been proven to improve mortality or neurological outcomes. The available alternatives are focused on the prevention of risk factors, managing complications following the initial hemorrhage, and considering surgical interventions in selected cases [6].

From a pathophysiological perspective, both IS and HS involve a complex series of events known as the ischemic cascade. Among these, the inflammatory and immune responses play a crucial role in brain damage during the acute phase, which lasts for hours to days following the onset of the stroke [7]. In the case of HS, the presence of blood and its components in the cerebral parenchyma aggravates the condition by causing mass effects and hematoma formation, which further worsen the patient’s prognosis [8]. After the acute phase, many stroke patients, both in experimental and clinical settings, may develop systemic immunosuppression (SI). This SI is primarily driven by the massive release of catecholamines (mainly norepinephrine), corticosteroids, and anti-inflammatory cytokines, resulting from overactivation of the sympathetic nervous system (SNS) and the hypothalamic–pituitary–adrenal (HPA) axis. These dysregulations in communication between the injured brain and the immune system contribute to the development of complications such as urinary tract infections (UTI) and stroke-associated pneumonia (SAP), which occur in 30–60% of stroke patients, impairing recovery and increasing mortality rates [9]. Although it has been shown that this disruption in communication between the brain and the immune system, along with factors such as the initial size of the lesion (rather than its location) [10,11], patient dysfunctions, and hospital management, contribute to the development of stroke-associated infections (SAIs), the precise origin of these infections is not yet fully understood. Recent scientific evidence, however, has emerged at both experimental and clinical levels, suggesting that alterations in the gut microbiota, damage to the intestinal barrier, and the subsequent translocation of bacteria from the gut to other organs may be additional factors that contribute to the development of infections following stroke [12].

Given the high prevalence and significant impact of SAIs on stroke prognosis and mortality, along with the emerging role of gut dysbiosis, gastrointestinal (GI) barrier disruption, and bacterial translocation (BT) as potential contributors to SAI development, this review will summarize recent advances in understanding these processes. It will also discuss their implications for SAI development and explore future perspectives for addressing these complications to improve stroke treatment and patient outcomes.

## 2. Microbiota–Gut–Brain Axis, Intestinal Dysbiosis, and Gut Barrier Damage After IS

The human microbiota is composed of a diverse array of microorganisms that inhabit various surfaces and mucosal membranes of the body. Among these, the intestinal microbiota hosts the largest and most diverse community of microbes, including bacteria, archaea, eukarya, viruses, and parasites [13]. This review focuses primarily on the bacterial component of the intestinal microbiota for two key reasons: first, it is the most extensively studied community, and second, there is substantial evidence supporting its role in both healthy and pathological states. The gut environment is conducive to the growth of bacteria from seven predominant phyla: *Firmicutes*, *Bacteroidetes*, *Actinobacteria*, *Fusobacteria*, *Proteobacteria*, *Verrucomicrobia*, and *Cyanobacteria*. Of these, *Bacteroidetes* and *Firmicutes* account for over 90% of the total bacterial population [14]. Under normal physiological conditions, the gut microbiota plays a critical role in host immune system development and regulation, serves as a source of nutrients, maintains metabolic homeostasis, and influences the function of distant organs, including the central nervous system (CNS). In this sense, it has established the microbiota–gut–brain axis (MGBA), which is a bidirectional communication system that links the gut, microbiota, and brain, allowing for a reciprocal exchange of signals. This system operates through the nervous and circulatory systems, with various biomolecules, transmitters, and hormones traveling between these components to maintain proper function and homeostasis. Communication within the MGBA occurs via both neuronal and non-neuronal mechanisms, involving two primary pathways: (1) Top-down (brain-to-gut) signaling: In this pathway, the brain communicates with the gut through the autonomic nervous system (ANS), including both parasympathetic (PNS) and sympathetic nervous system (SNS) fibers. These neural signals can also influence the enteric nervous system (ENS), a network of neurons located in the gut wall’s submucosa and myenteric plexi. These inputs regulate gut motility, permeability, microbiota composition, and immune cell activation. Additionally, the HPA axis plays a critical role in mediating the brain’s response to stress and modulating gut functions; (2) Bottom-up (gut-to-brain) signaling: This pathway conveys signals from the gut to the brain through several mechanisms. The vagus nerve (VN), which is composed of 80% afferent (gut-to-brain) and 20% efferent (brain-to-gut) fibers, serves as a key route for communication. Afferent fibers are activated by microbial compounds, metabolites, and hormones (e.g., serotonin, cholecystokinin, glucagon-like peptide-1, and peptide YY) released from enteroendocrine cells in the gut epithelium. These signals activate brain regions such as hypothalamic neurons that regulate pituitary secretions, as well as the nucleus tractus solitarius and its downstream projections. Additionally, immunogenic endotoxins, such as lipopolysaccharides (LPS), can trigger neuroinflammation by directly activating brain cells or through peripheral immune cell migration to the brain. Furthermore, the microbiota produces a variety of metabolites—including neurotransmitters, short-chain fatty acids (SCFAs; acetate, butyrate, propionate), indoles, and secondary bile acids—that are believed to enter the bloodstream and reach the brain, modulating the function of neurons, microglia, astrocytes, and the blood–brain barrier (BBB) [15,16,17,18,19].

**Figure 1:**
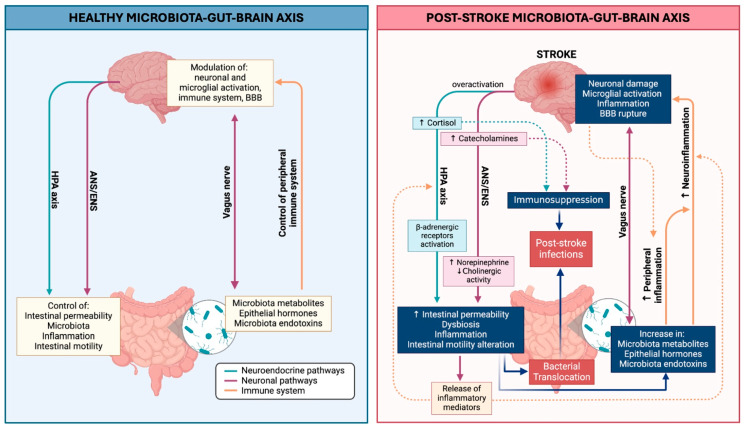
Schematic representation of the MGBA under two distinct conditions. Left: The primary pathways involved in the MGBA in a healthy state, illustrating the connections between the brain and gut through neuroendocrine pathways (ANS and ENS, including the vagus nerve, and the HPA axis), as well as the immune system. Right: Alterations in MGBA pathways following stroke. The increase in catecholamines and cortisol induces systemic immunosuppression. Stroke-induced overactivation of the HPA axis and the ANS/ENS systems leads to increased gastrointestinal permeability, dysbiosis, inflammation, and alterations in intestinal motility. Together, these factors contribute to the exacerbation of both peripheral and brain inflammatory processes, as well as an increased susceptibility to SAIs by promoting BT from the gut to sterile organs, thereby raising the risk of secondary infections.

In neurodegenerative diseases, including stroke, alterations in MGBA communication have been implicated in the progression of associated pathological conditions [20,21]. Specifically, after a stroke, three key failures within the MGBA contribute to the disruption of gut–brain communication: (1) impaired neurotransmission inside the ANS, (2) dysregulation of the HPA axis, and (3) immune system dysfunction, which alters the composition of the gut microbiota and compromises the integrity of the intestinal barrier (Figure 1).

### 2.1. Impaired Neurotransmission Inside the ANS

It is important to remember that the ANS, including its SNS and PNS divisions, along with the ENS, are the primary components of the brain-to-gut pathway. These systems innervate various gastrointestinal cells, blood vessels, and glands, modulating gastrointestinal motility, permeability, immune cell activation, secretions, and the composition of the gut microbiota [22]. Following brain injury, overactivation of the SNS leads to excessive norepinephrine release and a reduction in cholinergic activity within the intestinal neuronal pathways. These changes are associated with decreased intestinal motility, reduced mucin secretion, and disruption of the gut barrier, as observed in experimental stroke. In fact, GI dysfunctions such as dysmotility and constipation are experienced by approximately 50% of stroke patients [23]. A study by Stanley et al. demonstrated that after experimental IS, an increased ratio of adrenergic to cholinergic neurons and altered levels of catecholamines in the GI tract were linked to damage of the GI mucosal barrier [24]. Another study found that stroke-induced dysregulation of mucoprotein production, a reduction in mucus-producing goblet cells, and changes in cecal bacterial communities were correlated with the severity of brain injury, highlighting the impact of ANS dysregulation on intestinal motility after stroke [25]. In fact, the impaired formation of the mucus layer after stroke may allow gut commensal bacteria to come into direct contact with the intestinal epithelium, triggering immune activation, bacterial translocation, and potentially leading to post-stroke infections [24,26,27]. Additionally, it has been shown that paralysis of the ileum alone can induce microbiota changes, suggesting CNS-mediated dysbiosis [28]. On the other hand, within the gut-to-brain communication pathway of the MGBA, the VN plays a crucial role in relaying information from the GI system to the brain. This communication is influenced by microbial metabolites recognized by the intestine. After stroke, changes in microbial metabolite production caused by gut dysbiosis can alter gut–brain signaling via the VN, directly impacting brain function [29]. Despite these findings, which clearly demonstrate how post-stroke alterations in neural circuits between the brain and gut affect both the GI environment and brain function, much of the evidence comes from experimental studies. Clinically, while there is strong data showing dysregulation of the ANS and its impact on stroke outcomes [30,31], further research is needed to understand its role in GI dysbiosis and gut barrier disruption.

### 2.2. Dysregulation of the HPA Axis

Regarding the neuroendocrine system, one of the key pathways affected by stroke is the HPA axis, whose overactivation plays a critical role in the stress response. This overactivation occurs in response to vagal afferent fibers during a stroke, triggering the release of cortisol from the adrenal cortex [32]. However, there is currently insufficient evidence to support the hypothesis that elevated cortisol levels directly cause an imbalance in the gastrointestinal ecosystem in the context of stroke. That said, stress—a common pathological condition among stroke patients—can increase intestinal permeability through mast cell activation mediated by corticotropin-releasing hormone (CRH) [33]. Additionally, the HPA axis, through the stimulation of β-adrenergic receptors of the SNS, contributes to increased gastrointestinal permeability, promotes the generation of Th17 cells in the gut, and induces changes in the composition of the gastrointestinal microbiota in response to stress [34,35]. These changes in gastrointestinal permeability and microbiota composition trigger the release of inflammatory mediators, including pro-inflammatory cytokines, microbial antigens, and prostaglandins, capable of crossing the blood–brain barrier (BBB) and activating the HPA axis [36]. These alterations in permeability and microbiota composition can trigger the release of inflammatory mediators, including pro-inflammatory cytokines, microbial antigens, and prostaglandins, which have the potential to cross the blood–brain barrier (BBB) and further activate the HPA axis. These findings suggest that the HPA axis plays a role in bidirectional communication within the MGBA. However, more research is needed to confirm the relationship between HPA axis activation and gastrointestinal dysbiosis following stroke.

### 2.3. Failures in the Immune System

Following a stroke, local inflammation within the damaged brain (neuroinflammation) begins immediately after the arterial occlusion or rupture. During the acute phase, microglia, the resident innate immune cells, are rapidly activated. This inflammation also disrupts the blood–brain barrier (BBB), leading to the release of damage-associated molecular patterns (DAMPs) from dying cells into the peripheral circulation. This process triggers the activation and infiltration of peripheral immune cells into the injured brain [37]. As the stroke progresses from the acute to chronic phase, SI develops alongside ongoing neuroinflammation in the ischemic brain. Evidence suggests that in addition to immune system exhaustion resulting from the intense initial inflammation after stroke, excessive activation of the SNS and the HPA axis also contribute to SI. On one hand, activation of sympathetic neurons after stroke leads to the high secretion of catecholamines in various tissues, including lymphoid organs, and into the bloodstream from the adrenal medulla. Experimental studies have shown that overactivation of the SNS plays a key role in SI and post-stroke bacterial infections, primarily due to lymphopenia caused by excessive catecholamine release in immune organs. Moreover, β-receptor blockade using propranolol has been shown to reverse both lymphopenia and lymphocyte dysfunction, as well as reduce post-stroke infections. However, high doses of propranolol are required, and some studies report conflicting results, such as a recent investigation where low doses of propranolol did not reverse either the immunodepression or dysbiosis induced by experimental ischemic stroke [24,38,39,40,41]. On the other hand, the activation of the HPA axis also contributes to stroke-induced immunodepression. Key cytokines released from the ischemic brain, such as IL-1β and TNF-α, stimulate the paraventricular nucleus of the hypothalamus, which then triggers glucocorticoid release from the adrenal glands via corticotropin-releasing factor (CRF) and adrenocorticotropic hormone (ACTH). This release of glucocorticoids suppresses the production of pro-inflammatory cytokines while promoting the release of anti-inflammatory cytokines. Clinically, cortisol levels have been found to correlate not only with lymphopenia but also with stroke severity and the development of post-stroke infections [42,43]. In addition to the effects of the SNS and HPA axis on the immune system, alterations in hepatic function and the gastrointestinal (GI) tract have significant consequences for both local and systemic inflammation following stroke. Concerning the liver, overactivation of the ANS and the dysregulation of the HPA axis cause profound alterations in glucose metabolism, bilirubin levels, and liver enzyme activity, which further activate the immune system, directly impacting inflammation and damage in the ischemic brain [44,45,46]. The close communication between the liver and the intestine is well documented, and it has been demonstrated that liver function alterations due to stroke have a direct impact not only on GI immunity but also on intestinal microbiota homeostasis [47]. The GI tract itself serves as a crucial immune organ, housing more than 70% of the body’s immune cells. Therefore, disruptions in this system can affect the systemic immune response, particularly in recruiting immune cells to the ischemic brain. Stroke has been shown to induce pronounced GI dysbiosis, promoting the growth of opportunistic bacteria and reducing mucus secretion in the GI tract. This disruption brings immune cells into direct contact with altered microbiota molecules, triggering activation of various immune cell populations, particularly lymphocytes, which migrate from the GI tract to the affected brain. Experimental studies have demonstrated that post-stroke GI dysbiosis leads to increased trafficking of IL-17+ γδ T cells from the gut to the ischemic brain via the leptomeninges, while regulatory T cells decrease [28,48]. Recent research has shown that in addition to T cells, dendritic cells and macrophages also migrate to the brain and immune organs, such as the spleen and lymph nodes, following stroke [49]. Beyond the effects of GI dysbiosis on immune responses, reduced mucus secretion and intestinal barrier damage result in the release of pathogen-associated molecular patterns (PAMPs) into the systemic circulation. These are recognized by immune cell receptors and exacerbate the inflammatory response in stroke [12].

### 2.4. Clinical Evidence of Post-Stroke GI Dysbiosis and Leaky Gut

Although most evidence regarding GI dysbiosis following stroke comes from experimental studies, some clinical research also supports this process. One of the first signs of post-stroke dysbiosis is that approximately 50% of stroke patients report GI symptoms [23]. Additional clinical data come from analyses of the GI microbiota in fecal samples from stroke patients compared to healthy controls. These studies show that the phyla *Firmicutes* and *Bacteroidetes* are the predominant members of the human GI microbiota, a composition that aligns with findings in rodent models [21,50]. Moreover, these clinical analyses reveal a shift in microbial composition in stroke patients, with the extent of dysbiosis correlating with lesion severity [51,52,53]. Most studies suggest that stroke primarily disrupts the balance between *Firmicutes* and *Bacteroidetes*, with an increased abundance of *Firmicutes* acting as an independent predictor of ischemic stroke risk. Specifically, genera such as *Streptococcus*, *Lactobacillus*, and *Prevotella*, which belong to the *Firmicutes* phylum, are more prevalent in ischemic stroke cases [51,54,55,56]. However, other studies have shown an elevation in the phylum *Proteobacteria*, and in more severe cases, this increase is accompanied by a reduction in *Firmicutes* abundance [53]. For instance, a study by Haak et al. found an increased abundance of *Proteobacteria*, while levels of *Firmicutes* and *Bacteroidetes* were reduced in stroke patients [52]. Additionally, a recent study analyzing changes in the gut microbiota in stroke patients during the subacute and chronic phases (4 weeks post stroke) compared to healthy controls revealed a reduction in butyrate-producing bacteria, such as *Butyricimonas*, in stroke patients. This reduction is linked to increased GI permeability and inflammation. The study also observed at the subacute phase an increase in *Lactobacillus*, a benign bacterium, which may compensate for the reduction of butyrate-producing bacteria and potentially offer benefits after stroke. It was further demonstrated that post-stroke rehabilitation helps gradually restore microbiota populations to levels similar to those of healthy individuals [57]. Regarding α-diversity, an indicator of microbial diversity within a sample, some studies report lower microbial diversity in ischemic stroke cases compared to healthy controls [52,58], while others show significantly higher α-diversity in stroke patients [53]. Similarly, findings on β-diversity, which measures the diversity between sample groups, vary, with some studies showing significant differences between stroke patients and controls, while others report no change [52,53,54,55,58].

Finally, while there is evidence suggesting markers of GI barrier damage or permeability after stroke, the direct relationship between these markers and gut damage remains unclear. In other conditions, such as autism, stress, or metabolic syndrome, elevated levels of markers like lipopolysaccharide-binding protein (LBP) or soluble CD14 (sCD14) have been directly linked to the leaky gut process. In stroke, however, elevated levels of these proteins are primarily associated with the development of infections [59,60]. In these other conditions, in addition to the aforementioned markers, proteins like zonulin have proven to be reliable indicators of intestinal permeability [61,62,63], suggesting that more research is needed in stroke to explore their potential role in predicting future complications.

## 3. Bacterial Translocation After IS and Its Contribution to the Development of SAIs

As mentioned in the Introduction, 30–60% of stroke patients develop SAIs, which worsen prognosis and increase mortality, with UTIs and SAP being the most common. Several factors contribute to the development of these infections, including neurological dysfunctions such as dysphagia and bronchoaspiration, management during hospitalization (including the use of invasive catheters), and the SI that follows the acute phase of stroke [9]. However, recent experimental and clinical evidence suggests that bacterial translocation (BT) from the intestine to sterile organs, such as the lungs, may also play a role in the development of these infections. This section will explore experimental and clinical data on BT, its role in infection development, and its impact on stroke outcomes (see Table 1).

BT occurs due to GI dysbiosis and damage to the intestinal barrier that follows a stroke. Experimental evidence supporting this process has been available for several years. In 2009, our group identified BT to various peritoneal organs 24 h after experimental stroke, but only in animals subjected to subacute stress in the seven days preceding surgery. Furthermore, this stress reduced colonic IgA levels—an essential immunoglobulin for pathogen defense at mucosal surfaces—elevated intestinal inflammation, with larger infarct volumes, and worse functional outcomes in stroked animals with BT [70]. While experimental studies continued to explore this process, it was not until 2016 that more conclusive experimental and clinical evidence on BT after stroke emerged. One such study by Crapser et al. demonstrated SI, leaky gut, and BT in both young and aged animals following experimental stroke. Despite observing smaller infarct volumes in aged animals (18–20 months old), these animals exhibited higher mortality and more severe functional deficits at 72 h and 7 days after stroke. The study also assessed intestinal barrier integrity at 72 h using oral contrast agents (NaF and FITC-dextran) and found increased permeability and higher blood levels of lipopolysaccharide-binding protein (LBP) in both groups. Their findings confirmed that BT occurred in both young and aged animals, with bacterial growth observed in organs of the peritoneal cavity and lungs. The primary route of BT was through the mesenteric lymph nodes (MLN), and the bacteria found outside the intestines varied, with *Escherichia* species predominating in younger animals and *Enterobacter* species in older ones. Furthermore, stroke induced significant lymphopenia in peripheral blood, and older animals showed greater T lymphocyte infiltration in the ischemic brain 72 h post-surgery [26]. In another study published in the same year by Stanley et al., GI dysbiosis, leaky gut, and BT were again observed 24 h after experimental ischemic stroke, both in animal models and in blood, urine, and saliva samples from stroke patients. This study found that 22% of stroke patients with SAIs exhibited the growth of typical intestinal bacteria, such as *Enterococcus* spp., *Escherichia coli*, and *Morganella morganii*, whereas no culturable organisms were found in healthy or control patients. To corroborate these findings, the researchers subjected germ-free (GF) and specific pathogen-free (SPF) animals to experimental IS, revealing significant changes in the intestinal microbiota, a disruption in gut innervation, increased permeability of the intestinal barrier (peaking 3 h post-surgery), and BT to various organs, including the lungs. Notably, *Lactobacillaceae*, *Clostridiaceae*, and *Enterobacteriaceae* were found in the lungs of ischemic animals. In another experiment, where *Enterococcus faecalis* was administered to GF mice, the bacteria translocated to all analyzed organs in IS animals, further confirming the BT process [24].

In a study published in 2019, Wen et al. examined why older patients are more prone to secondary infections after IS. Using a transient IS model, the authors found that older animals (over 12 months old) developed more spontaneous bacterial lung infections than younger animals. The study also highlighted that older post-stroke mice exhibited increased intestinal inflammation and disrupted gut barrier integrity, marked by reduced expression of mucin and tight junction proteins. Elevated TNF-α levels in the colon of older mice promoted the translocation of bacteria to the lungs following stroke. These results again support the idea that age-related dysfunction of the GI barrier enhances BT, contributing to the increased risk of post-stroke infections [65]. Further evidence from Diaz-Marugan et al. (2023) demonstrated that IS induced lymphocytopenia and widespread colonization of the lungs and other organs by opportunistic gut commensal bacteria. This effect was linked to a compromised gut epithelial barrier, increased intestinal inflammation (including complement and NF-κB activation), reduced gut regulatory T cells, and shifts in gut lymphocyte populations toward γδ T-cells and T-helper 1/17 phenotypes. Stroke also caused a reduction in gut fermenting anaerobic bacteria, while *Enterobacteriaceae* species were expanded. Anti-inflammatory treatment with an NF-κB inhibitor completely prevented the overgrowth of *Enterobacteriaceae*, although it did not prevent post-stroke lung colonization [40]. Additionally, unpublished data from our group indicate that approximately 70% of rats subjected to experimental ischemic stroke developed BT, with bacteria translocating to various organs, including the lungs. These animals also exhibited signs of leaky gut, as determined by MRI and immunohistochemistry, and our findings suggest that BT contributed to increased infarct size 72 h post stroke. Notably, absence of the TLR4 receptor reduced infarct volume, decreased intestinal permeability, limited BT, and improved the inflammatory response after stroke.

However, some studies, like those by Oyama et al., did not observe signs of intestinal inflammation, leaky gut, or BT following experimental IS, despite detecting changes in tight junction protein expression in the ileum [64].

Finally, studies on Alzheimer’s, Parkinson’s, and autism models have shown that BT can occur from the gut to the brain via the VN, accelerating disease progression. These findings highlight the need to investigate the gut-to-brain translocation process in the context of stroke as well [71].

## 4. Intestinal Dysbiosis, Gut Barrier Damage, and Bacterial Translocation After HS

Within the pathophysiology of stroke, HS shares similar inflammatory and immune alterations as IS [8]. Moreover, patients with HS develop secondary infections in proportions comparable to those seen in IS. Despite these similarities, the research on alterations in the MGBA, GI dysbiosis, gut barrier damage, and BT following HS is still limited. Regarding MGBA alterations after HS, while changes in the ANS/ENS, hyperactivation of the HPA axis, and immune system changes are similar to those observed in IS, further investigation is necessary to clarify these processes in the context of HS [72]. Concerning GI barrier damage, GI dysbiosis, and BT after HS, Table 1 summarizes the few existing studies on this topic. A recent study by Cheng et al. demonstrated that experimental HS in mice rapidly led to gut mucosal destruction, delayed small intestinal motility, GI barrier dysfunction, and increased inflammatory and oxidative stress responses, with symptoms appearing as early as 2 h after HS and persisting up to 7 days. These findings suggest that HS may induce immediate and sustained damage to gut structure and barrier function, potentially associated with the upregulation of inflammation and oxidative stress markers [66]. Regarding GI dysbiosis following HS, experimental intracerebral hemorrhage (ICH) has been shown to cause gut microbiota dysbiosis, which in turn impacts ICH outcomes through immune-mediated mechanisms. HS also reduces microbiota diversity and promotes microbiota overgrowth, phenomena that may be involved in reduced intestinal motility and increased gut permeability. Furthermore, recolonizing ICH mice with a healthy microbiota ameliorates functional deficits and neuroinflammation after ICH. Cell-tracking studies have also demonstrated the migration of intestinal lymphocytes to the brain following ICH. Additionally, therapeutic fecal microbiota transplantation has shown improvement in GI barrier damage [67].

In the clinical setting, a study by Wang and colleagues analyzed fecal samples collected at 24, 72 h, and 7 days after HS, focusing on GI dysbiosis through 16S rRNA sequencing and its correlation with functional outcomes. The results revealed that the composition and diversity of gut microbiota in patients with ICH differed significantly from the control group and changed dynamically with the progression of cerebral hemorrhage. The abundances of *Enterococcaceae*, *Clostridiales incertae sedis XI*, and *Peptoniphilaceae* were significantly increased in ICH patients, while *Bacteroidaceae*, *Ruminococcaceae*, *Lachnospiraceae*, and *Veillonellaceae* were notably reduced. The relative abundance of *Enterococcus* gradually increased with the duration of ICH after hematoma evacuation, while the abundance of *Bacteroides* gradually decreased. The abundance of *Enterococcus* before surgery was negatively associated with neurological function prognosis, while the initial ICH score and *Lachnospiraceae* status were independent risk factors for predicting neurological outcomes after ICH [69].

Finally, evidence of BT following HS is limited to a single experimental study published thus far. This study examined changes in the immune response and GI barrier function at 24, 72 h, and 7 days, aiming to clarify the mechanism of secondary pulmonary infections after HS in mice. In addition to revealing alterations in immune response, signs of peripheral immunosuppression, and morphological changes with increased GI barrier permeability, the study also observed signs of lung disease and immune response changes, particularly at 7 days post HS. 16S rRNA sequencing of lung and ileum microbiota showed strong alterations after stroke, with microbial composition in both organs becoming similar by day 7, suggesting that intestinal bacteria migrated to the lungs and contributed to lung infection at this time point after HS [68]. In our laboratory, unpublished results also demonstrate the development of BT following experimental HS in rats, a process highly dependent on the initial volume of hemorrhage. This translocation of gut bacteria can even reach the lungs, altering the inflammatory response and impairing the neurological function of the animals. Additionally, our results indicate that the absence of TLR4 not only reduces hemorrhage size but also decreases BT processes.

## 5. Therapeutic Strategies That Could Help on Reducing Dysbiosis/BT and May Improve Stroke Outcomes

**Figure 2:**
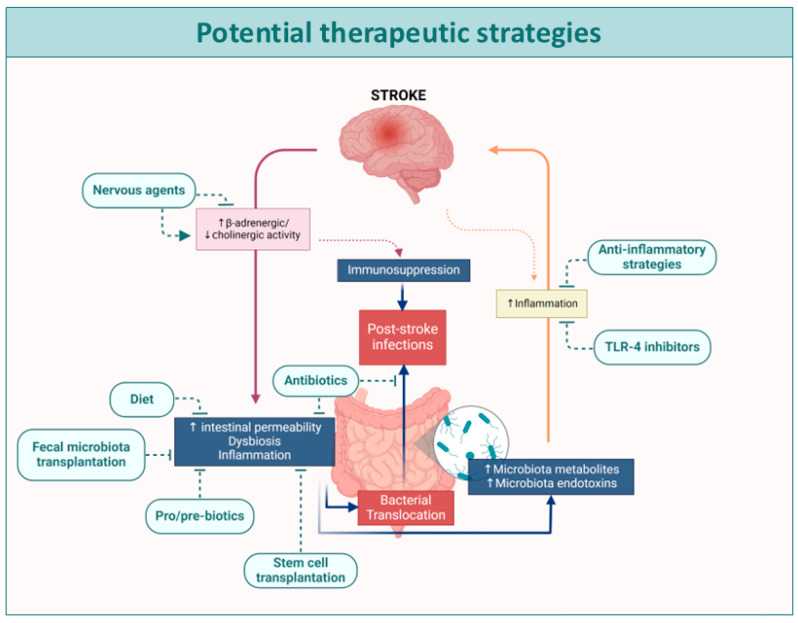
Potential therapeutic strategies to reduce GI dysbiosis, leaky gut, and BT after stroke.

As outlined in the introduction, current treatment options for stroke are limited, with no pharmacological therapies available for HS and only fibrinolytic treatment and/or mechanical thrombectomy for IS. In fact, approximately 70% of patients do not receive any form of treatment, highlighting the urgent need for new therapeutic strategies. Despite recent evidence linking alterations in the MGBA, GI dysbiosis, leaky gut, and BT to stroke, no therapies currently target gut dysbiosis or BT, nor their role in the development of SAIs. Given these findings and the potential for strategies that reduce gut dysbiosis/BT to improve stroke outcomes, this section will explore therapeutic approaches aimed at modifying intestinal dysbiosis/BT-related processes to prevent post-stroke complications (see Table 2 and Figure 2).

### 5.1. Antibiotics

Several clinical studies have examined the prophylactic and therapeutic use of antibiotics to prevent post-stroke infections and improve stroke outcomes. The results of these studies indicate that, while antibiotics have reduced overall infection rates, their use in stroke patients has not led to improvements in neurological function or mortality rates [73,74,75]. Although antibiotic treatment for acute stroke may not be recommended, it remains the only current option for treating SAIs [109]. Indeed, antibiotic therapy may help reduce infections caused by the translocation of gut bacteria to sterile organs following disruption of the GI barrier; however, antibiotic use may also negatively affect this barrier.

Similarly, experimental stroke studies involving antibiotic therapy have yielded contradictory results. On the one hand, some studies have demonstrated beneficial anti-inflammatory effects associated with changes in the microbiota following antibiotic treatment [48,76,77]. On the other hand, Winek et al. (2020) showed that antibiotics could disrupt the composition and homeostasis of the intestinal microbiota, potentially increasing the risk of infection through the production of bacterial fragments [27].

### 5.2. Dietary Interventions

The importance of a healthy diet in stroke prevention is well established, as it has been shown that the development of key stroke risk factors such as diabetes, hypertension, and obesity is closely linked to dietary patterns [110]. Furthermore, the impact of these risk factors on the induction of GI dysbiosis has been clinically demonstrated [15]. Therefore, dietary interventions may serve as a therapeutic strategy to prevent BT in stroke patients by modulating gut homeostasis, barrier integrity, and inflammatory processes [111].

Specific diets.

The Mediterranean diet (MD), which is rich in vegetables, legumes, olive oil, and low in meat, has been strongly associated with a reduction in stroke risk factors and incidence [112]. Studies have shown that the MD promotes an anti-inflammatory gut microbiota by increasing *Bacteroidetes* species and decreasing the *Firmicutes/Bacteroidetes* ratio [113]. In cardiovascular and cerebrovascular patients, the MD enhances beneficial *Bacteroidetes* while depleting harmful *Firmicutes*, compared to a Western diet [114]. Additionally, MD has been shown to increase SCFAs and decrease trimethylamine N-oxide (TMAO) production, improving gut–brain communication and reducing gut inflammation and barrier damage [114]. Similarly, following a vegetarian diet for a short period has been shown to increase butyrate-producing bacteria and enhance GI microbiota diversity, both of which are beneficial for stroke recovery [115]. In contrast, high salt and sugar consumption disrupts gut flora diversity and triggers inflammatory responses. Excessive salt intake has been clinically linked to a reduction in *Lactobacillus* species and increased T cell activity in humans [116]. Experimentally, a high-salt diet in mice has been shown to cause neurovascular alterations, inflammation, and cognitive impairment, driven by intestinal activation and migration of IL-17 T cells from the gut to the brain [117]. Likewise, sugar- and fat-rich diets in rats alter gut microbiota, particularly the *Bacteroidetes* phylum [118]. On the other hand, low-protein and calorie-restricted diets have demonstrated neuroprotective and anti-inflammatory benefits by restoring gut microbiota [78] and increasing *Bifidobacterium* species, respectively [79].

Short-chain fatty acids (SCFAs) and fiber.

SCFAs, primarily acetate, propionate, and butyrate, are products of bacterial fermentation of dietary fiber in the colon. They are produced by gut commensal bacteria, including *Lactobacillus*, *Bacteroides*, *Firmicutes*, *Prevotella*, *Clostridium*, and *Bifidobacterium* [119]. In stroke patients, lower levels of SCFAs and SCFA-producing bacteria have been associated with increased stroke severity, poor prognosis [120,121], and post-stroke infections [52], highlighting their potential as therapeutic targets. Moreover, the absence of SCFAs in feces has been linked to cognitive impairment following stroke [122]. Experimentally, SCFA supplementation in mice has shown improvements in recovery, cortical reorganization [80], reduced inflammation, and oxidative stress [123,124,125], while preserving GI barrier integrity [80,126,127]. Additionally, a high-fiber diet containing inulin increased SCFA production and promoted an anti-inflammatory microglial profile [128]. Thus, modulating SCFAs or SCFA-producing bacteria may represent a promising approach to prevent GI dysbiosis and BT after stroke.

Trimethylamine N-oxide (TMAO).

TMAO metabolites, produced by intestinal microbiota from L-carnitine and choline (both abundant in meat), are associated with atherosclerosis, thrombosis, and stroke [129,130]. Antibiotic treatments reduce TMAO production, but levels rebound after treatment [131]. Diet also influences TMAO levels, while high-fat and ketogenic diets tend to increase their production [132], and the MD diet has been shown to reduce TMAO levels [133]. Moreover, stroke patients exhibit lower TMAO levels and altered intestinal microbiota, which may be explored in future studies [53].

### 5.3. Fecal Microbiota Transplantation

Fecal microbiota transplantation (FMT) involves transferring stool from a healthy donor to restore the gut bacterial balance and GI homeostasis, thereby repairing GI barrier damage and permeability caused by various diseases, including neurological disorders [134,135]. Experimental studies have demonstrated the beneficial effects of FMT on stroke outcomes [28,51,136], particularly by promoting the growth of *Lactobacillus*, increasing IL-10 release, and enhancing T regulatory cells, which improve the GI environment, prevent gut leakage, and reduce gut inflammation [81,82,83,137]. While the exact mechanisms remain unclear, restoring gut homeostasis appears to be critical, suggesting that the beneficial effects observed in gut dysbiosis could potentially be extended to BT-related processes.

### 5.4. Probiotic and Prebiotic Agents

Probiotics are live microorganisms, often referred to as “good” or “friendly” bacteria, that provide health benefits when consumed in adequate amounts. They are commonly found in fermented foods and dietary supplements. In contrast, prebiotics are non-digestible food components, such as fibers, that promote the growth and activity of beneficial bacteria in the gut. Prebiotics serve as a food source for probiotics and other healthy gut microbiota, helping to maintain a balanced and thriving gut ecosystem [138]. These nutritional supplements have been shown to improve gut microbiota homeostasis and GI barrier integrity in stroke patients [139], potentially preventing BT-related processes as well. Specifically, therapy with *Lactobacillus* and *Bifidobacterium* maintained GI barrier integrity and prevented diarrhea in stroke patients [84]. Additionally, a small clinical study demonstrated that probiotics improved post-stroke cognitive impairment [122], highlighting the importance of gut homeostasis for the proper functioning of the MGBA. Experimental studies in stroke models have shown that probiotics produce an anti-inflammatory and antioxidant response by suppressing TNF-α and free radical production through the modulation of TLR activation in the gut [85,140]. Notably, the administration of *Clostridium butyricum* exhibited antioxidant and anti-apoptotic effects in this context [86]. Regarding the beneficial effects of prebiotic administration, it has been shown that treatment with lactulose promotes intestinal barrier repair and reduces inflammation [87], while resveratrol modulates inflammation by regulating the intestinal T cell response [88]. Additionally, indole-3-propionic acid (IPA) reduces harmful bacteria, regulates T-reg cell response, and maintains gut barrier integrity [89]. Furthermore, in clinical settings, prebiotics can influence the production of SCFAs, thereby regulating the local inflammatory response in the gut by stimulating phagocytosis by macrophages [141]. Another clinical study reported that treatment with the prebiotic barley, one of the oldest cereal grains cultivated, increased butyric acid-producing bacteria, thus elevating its levels in the gut [142]. More interestingly, administration of prebiotics has been shown to decrease both the incidence of SAP in patients admitted to intensive care units and the length of hospital stays [143].

### 5.5. ANS Modulators: β-Adrenergic Blockers and Cholinergic Agonists

After stroke, there is an imbalance between cholinergic and adrenergic signaling, which leads to immunosuppression and SAI development [90,144]. Therefore, therapeutic approaches targeting the signaling pathways of these neurotransmitters might be another strategy to prevent BT-associated complications. The clinical use of β-adrenergic blockers in stroke patients is controversial since no clear benefits in the final outcomes have been observed [145,146]. On the one hand, it has been demonstrated that the use of propranolol and other β-adrenergic blockers reduced the incidence of SAIs, although to achieve this effect required the administration of very high doses of these drugs [90,91]. Experimentally, β-blockers also showed a decreased BT from the gut to the lungs, liver, and spleen and prevented bacterial infections after stroke [24,92,93,94]. But, in contrast, in a recent study, it was found that low doses of propranolol were unable to counteract the immunosuppression and dysbiosis triggered by ischemic stroke [24,38,39,40,41]. In the context of the use of cholinergic agonists, neuroprotective and anti-neuroinflammatory effects have been described in the field of experimental stroke, and a reduction of GI permeability after experimental traumatic brain injury [95,96,97].

### 5.6. Stem Cell Transplantation

Another emerging strategy gaining attention in the field of stroke is focused on repairing the GI barrier through stem cell therapy, with the aim of preventing leaky gut and BT induced by this condition [147]. In this regard, Mani et al. demonstrated that intestinal epithelial stem cell (IESC) transplants can prevent gut permeability and reduce levels of LPS and IL-17 after experimental stroke [98]. Further clinical and experimental investigations into this therapy are needed to provide more insight into the potential use of stem cell transplants in modulating BT after stroke.

### 5.7. Anti-Inflammatory and Immunomodulatory Strategies

BT results from the inflammatory response following a stroke, which activates the intestinal immune system and increases gut permeability. Therefore, targeting immune cells and inflammatory cytokines could help reduce BT after a stroke. Anti-inflammatory approaches that target neutrophils, macrophages, and T cells [100,101,105], inhibition of cytokines such as IL-6, IL-17, and TNF-α [102,106,107], or modulate T-reg cells [103], have shown protective effects in experimental stroke, preventing GI barrier damage and permeability associated with BT. Specifically, experimental studies have described the role of TNF-α in gut bacterial dissemination and microbiota alterations after stroke [65,148]. However, the use of immunomodulatory strategies may lead to SI, contributing to post-stroke infections [149].

Stroke-induced alterations in the GI barrier can result in the release of endotoxin LPS into the bloodstream, which amplifies inflammation through TLR4 activation and exacerbates brain damage [150,151,152]. Experimental studies have shown an association between GI dysbiosis and increased LPS levels in plasma after stroke [123,153], which can be prevented by antibiotic or stem cell therapy [76,98]. Interestingly, LPS preconditioning may have protective anti-inflammatory effects by increasing B regulatory cells after stroke [154].

Another strategy to counteract LPS-mediated effects involves the inhibition of TLR4. Preliminary results from our laboratory show that the absence of TLR4 reduces infarct volume, GI damage, and BT following experimental IS and HS. Additionally, a specific TLR4 aptamer (ApTOLL) has demonstrated neuroprotective and anti-inflammatory effects in experimental studies [104,108] and has shown safety and biological effects in a Phase Ib/IIa clinical trial with patients suffering from IS [99]. However, further studies are needed to assess its potential in treating BT-related processes.

In summary of this section, although promising evidence supports the therapeutic potential of these strategies in stroke, further experimental and clinical studies are necessary to clarify their specific effects on processes like GI dysbiosis, leaky gut, and BT particularly to prevent post-stroke complications such as SAIs. However, an important limitation must be considered: studies specifically focused on BT remain scarce. Most of the existing literature addresses dysbiosis and alterations in the gut microbiota, but these findings could also extend to processes involving BT.

## 6. Summary and Conclusions

In this review, we summarize the latest findings on how stroke affects communication between the brain and the gut by altering the microbiota–gut–brain axis (MGBA), with a particular focus on bacterial translocation (BT) as a potential cause of complications following stroke, such as the development of SAIs. Stroke, with the brain at its center, leads to dysfunctions at multiple levels, including the ANS, the HPA axis, and the immune system. First, the primary dysfunctions in the nervous pathways involve overactivation of the ANS, resulting in altered release of neurotransmitters such as norepinephrine and acetylcholine, which ultimately disrupt GI homeostasis and contribute to the development of systemic inflammation. Regarding the HPA axis dysfunctions, these primarily occur through excessive release of glucocorticoids at the GI level, which not only induces GI dysbiosis but also contributes to the development of the leaky gut process. Moreover, this massive release of glucocorticoids is another trigger of SI, which further contributes to the development of post-stroke infections. Additionally, neuronal death and BBB damage lead to the release of DAMPs into circulation. These DAMPs activate peripheral immune cell populations, promoting their infiltration into the brain and altering/activating the immune response in the gut. This immune activation not only facilitates the migration of lymphocytes from the gut to the affected brain but also plays a key role in dysbiosis and GI barrier permeability. Furthermore, all these MGBA alterations caused by stroke promote the migration of bacteria from the gut to sterile organs, a process known as BT. This review highlights the process of BT, summarizing the most recent experimental and clinical studies on IS and HS. Experimental studies provide substantial evidence demonstrating the occurrence of BT, particularly after IS, its impact on peripheral and central inflammation, and how this phenomenon could contribute to the development of secondary infections. However, it is important to note that in experimental studies, IS/HS is typically induced through surgery, with most studies using male animals that are free of comorbidities. Additionally, while these experimental studies show parallels to human conditions, differences in the baseline composition of the microbiota should be considered, as they may influence the processes of GI dysbiosis, leaky gut, and BT developed after stroke. Clinically, although there are fewer studies compared to experimental models, GI dysbiosis, leaky gut, and, more notably, BT have also been observed. Moreover, it has been demonstrated that BT may serve as another contributing factor to the development of SAIs.

Finally, although further research is needed, particularly experimental studies, a limited number of clinical findings have been presented regarding therapeutic strategies that may help mitigate the effects of GI dysbiosis, leaky gut, and BT following a stroke. Among these strategies highlighted by the available clinical data, the most promising for reducing these complications post stroke appear to be probiotics/prebiotics, agents that modulate ANS activity, and anti-inflammatory drugs such as ApTOLL.

## Figures and Tables

**Table 1:** Relevant experimental/clinical studies demonstrating the processes of GI dysbiosis, leaky gut, and BT after IS and HS.

BT AFTER IS				
Authors/Ref. Number	Year	Type	Model	Main Findings
Crasper et al. [26]	2016	Experimental	Male C57Bl/6 mice, aged 8–10 weeks and 18–20 months, subjected to 60 min transient MCAO (tMCAO) using the intraluminal model.	Demonstration of leaky gut and BT in young and aged animals following IS. Aged mice exhibited higher mortality, poorer functional outcomes, and differences in bacterial species found outside the gut compared to their younger counterparts.
Stanley et al. [24]	2016	Experimental/Clinical	7–10-week-old male C57BL/6J mice housed under specific-pathogen-free (SPF) conditions and germ-free (GF) 7-week-old male C57BL/6J mice subjected to 60 min tMCAO using the intraluminal model. A prospective cohort study conducted with 36 patients from Foothills Medical Centre, affiliated with the University of Calgary.	Experimentally, both GF and SPF mice exhibited significant GI dysbiosis, an imbalance in gut adrenergic/cholinergic innervation, increased intestinal permeability, and BT 24 h after IS. 22% percent of the cohort of patients with IS and SAIs exhibited growth of typical gut bacteria in blood, urine, and saliva samples.
Oyama N. et al. [64]	2018	Experimental	12-week-old male C57Bl/6J mice subjected to 60 min tMCAO using the intraluminal model.	Mice subjected to IS, despite detecting changes in tight junction protein expression in the ileum, no signs of intestinal inflammation, leaky gut, or BT were observed.
Wen et al. [65]	2019	Experimental/Clinical	Male C57BL/6J mice, aged 7–10 weeks and 12–15 months, subjected to 20 min tMCAO using the intraluminal model. Logistic regression modeling was used to determine the association between stroke and infection in a cohort of 509 patients from Monash Medical Centre.	Aged mice showed a higher number of bacterial lung infections, elevated gut inflammation, and leaky gut after IS compared to their young counterparts. Multivariable regression model of risk factors analysis showed that age, stroke severity, and the use of an indwell-ing catheter had a significant association with post-stroke infection and hence are independent predictors.
Diaz-Marugan et al. [40]	2024	Experimental	Male C57BL/6J mice aged 10–11 weeks and male and female Cx3cr1eGFP/+ mice subjected to 45 min tMCAO using the intraluminal model.	IS induced lymphopenia, intestinal permeability, GI dysbiosis, and altered gut inflammation 48 h after surgery. Additionally, IS caused BT to the peritoneal organs and lungs at this time point.
**DYSBIOSIS, LEAKY GUT** **, AND BT AFTER HS**				
Authors/Ref. Number	Year	Type		Main Findings
Cheng et al. [66]	2018	Experimental	Male C57BL/6J mice aged 6–8 weeks subjected to ICH by the autologous blood infusion model.	HS caused destruction of the gut mucosa, impaired small intestinal motility, increased intestinal permeability, and elevated gut inflammation and oxidative stress. These effects began as early as 2 h after HS and persisted for 7 days.
Yu X. et al. [67]	2021	Experimental	Male C57BL/6J mice aged 10–12 weeks subjected to HS by the collagenase model.	The GI dysbiosis found in HS mice, also affected the outcome through immune-mediated mechanisms. FMT from healthy/young mice to HS mice reduced the GI barrier damage.
Zhang et al. [68]	2021	Experimental	Male C57BL/6J mice aged 6–8-weeks subjected to HS by the collagenase model.	HS in mice induced changes in the immune response, signs of small intestine damage, morphological gut alterations, and increased intestinal permeability from 3 to 7 days after surgery. Similar dysbiosis was observed in both the ileum and lungs at 7 days, suggesting BT from the gut to the lungs following HS.
Wang et al. [69]	2024	Clinical	A study involving two cohorts: 30 control participants and 51 HS patients admitted to the emergency department of Zhengzhou Central Hospital, affiliated with Zhengzhou University, between May and September 2023.	Patients with HS exhibited significant differences in the composition and diversity of their GI microbiota compared to healthy subjects. These alterations changed dynamically over the course of HS, suggesting that GI dysbiosis could serve as a predictor of disease progression.

**Table 2:** Relevant clinical and experimental evidence regarding potential therapeutic strategies that might reduce GI dysbiosis/BT and improve stroke outcome.

Clinical		Experimental	
**ANTIBIOTICS**			
Ischemic stroke trials: PASS (Nº patients = 2225) [73], STROKE-INF (Nº patients = 1224) [74], and STRAWINSKI (Nº patients = 227) [75].	Reduction of overall infection rates but not improvements in stroke outcome.	Male and female C57Bl/6 and *db/db* mice aged 7–8 weeks subjected to 35 and 60 min tMCAO using the intraluminal model [27,48,76]. Male Wistar rats aged 4 weeks subjected to MCA occlusion using the endothelin-1 model [77].	Beneficial anti-inflammatory effects related to microbiota modifications [48,76,77]. GI dysbiosis increases the risk of infection through the production of bacterial fragments [27].
**DIETARY INTERVENTIONS**			
-	-	Male and female C57Bl/6 mice aged 6–10 weeks subjected to 30 and 60 min tMCAO using the intraluminal, and pMCAO photothrombotic and electrocoagulation models [78,79,80].	Beneficial anti-inflammatory effects, restoring gut microbiota after protein and caloric diet restrictions [78,79]. Preserved gut barrier integrity with butyric components [80].
**FECAL MICROBIOTA TRANSPLANTATION**			
-	-	Male and female C57Bl/6 mice aged 10–12 weeks and 16–18 months subjected to 60 min tMCAO using the intraluminal and pMCAO electrocoagulation models [81,82]. Male Sprague Dawley rats aged 9 weeks subjected to 120 min tMCAO using the intraluminal model [83].	Increased *Lactobacillus* [83] and T-regs cells [81,82] production, reducing gut leakage and gut inflammation after FMT.
**PROBIOTIC AND PREBIOTIC AGENTS**			
Ischemic stroke study (Nº patients = 60) [84].	Maintenance of GI barrier integrity and diarrhea prevention with *Lactobacillus* and *Bifidobacterium* treatment.	Male C57Bl/6 and ICR mice aged 6–10 weeks subjected to 45, 60, and 90 min tMCAO using the intraluminal, 20 min BCCAO using vascular clips, and pMCAO photothrombotic models [85,86,87,88,89].	TNF-α and free radical suppression through modulation of gut TLRs [85]. Antioxidant and anti-apoptotic effects by *Clostridium butyricum* treatment [86]. GI barrier repair and decreased inflammation after lactulose treatment [87]. Regulated intestinal T cell response by resveratrol [88]. Reduced harmful bacteria, regulated T-reg response, and maintained GI barrier integrity by IPA [89].
**ANS MODULATORS**			
Virtual International Stroke Trials Archive [90]. Historical cohort study with ischemic or hemorrhagic stroke patients (Nº patients = 625) [91].	Reduced urinary [90] or lung post-stroke infections after β-blockers treatment [91].	Male and female C57Bl/6 and Balb/c mice aged 7–12 weeks subjected to 30 and 60 min tMCAO using the intraluminal and TBI models [24,92,93,94,95,96]. Male Sprague Dawley rats aged 7 weeks subjected to 120 min tMCAO, using the intraluminal model [97].	Decreased BT from the gut to the lungs, liver, and spleen [24] and prevented bacterial infections after β-blockers [92,93,94]. Anti-inflammatory effects [95,96] and prevented GI permeability after cholinergic agonist treatment [97].
**STEM CELL TRANSPLANTATION**			
-	-	Male and female Sprague Dawley rats aged 5–7 and 12 months subjected to pMCAO by the endothelin-1 model.	Reduced GI permeability and LPS/IL-17 levels after IESCs transplant [98].
**ANTI-INFLAMMATORY STRATEGIES**			
Ischemic stroke trial (Nº patients = 151) [99].	ApTOLL showed excellent safety and reduced mortality and 90-day disability after treatment.	Male C57Bl/6 and ICR mice aged 6–15 weeks subjected to 20, 45, 60, and 90 min tMCAO using the intraluminal, and pMCAO using ligature models [40,65,100,101,102,103,104]. Male and female Sprague Dawley and Wistar rats aged 96 h and 5–12 weeks subjected to pMCAO, 120 min tMCAO using the ligature, and 45 and 60 min tMCAO using the intraluminal models [104,105,106,107,108].	Anti-inflammatory effects targeting neutrophils [105], macrophages [100], T cells [101], IL-6 [106], IL-17 [102], TNF-α [107], or modulating approaches of T-regs [103]. Reduced bacterial dissemination and dysbiosis targeting TFN-α [65]. Reduced stroke-induced gut overgrowth of *Enterobacteriaceae* after NF-κB inhibition [40]. Neuroprotective and anti-inflammatory effects displayed by ApTOLL [104,108].
